# Bibliographical Notices

**Published:** 1844-07-27

**Authors:** 


### BIBLIOGRAPHICAL NOTICES.

*The Cyclopædia of Anatomy and Physiology.* Edited by Robert B. Todd, M. D., F. R. S., &c. &c. Parts I to XXV. London.

*The Cyclopædia of Practical Surgery.* Edited by W. B. Costello, M. D. London.

*A Dictionary of Practical Medicine, comprising General Pathology, the Nature and Treatment of Diseases, Morbid Structures, &c. &c.* By James Copland, M. D., F. R. S., &c. Parts I to IX. London.

*The Cyclopædia of Practical Medicine.* Edited by John Forbes, M. D., F. R. S., Alexander Tweedie, M. D., F. R. S., and John Conolly, M. D. Revised, with additions, by Robley Dunglison, M. D. Parts I to IX. Philadelphia.

*Dictionnairé de Médecine ou Répertoire Général des Sciences Médicales, considérées sous le Rapport Théorique et Pratique.* Par MM. Adelon, Béclard, Bérard, &c. &c. Tome I—XXVIII. Paris.

*Dictionnairé des Etudes Médicales Pratiques.* Par MM. Amussat, Andral (A.), Mme. Boivin, &c. Tome I—IV. Paris.

*Encylopädisches Wörterbuch des Medicinischen Wissenschaften.* Herausgegeben von der Professoren der medicinischen Facultat zu Berlin. U. S. W. Band I—XXXI. Berlin.

It has seemed to us, that it would be interesting to many of our readers to have a brief notice of different encyclopædiac works on medical science, which either are, or ought to be, in course of publication at this time, in order that they may be able to form some idea of the progress of such extensive undertakings, and be enabled to judge of the value which they individually possess.

1. The "*Cyclopædia of Anatomy and Physiology*" was commenced in June, 1835; and has been nine years in reaching the 25th part, which considers the Muscles and Regions of the Neck, Nerve, Nervous System and Nervous Centres:—the first article by J. Simon, Esq., the second by Dr. Todd, the third by J. Anderson, Esq., and the fourth by Dr. Todd. In an advertisement prefixed to this part, which appeared in January, 1844, the Editor alludes to the difficulties he had experienced in issuing the parts regularly; and states, that "these obstacles, he is thankful to be able to say, no longer exist;" and on the cover of this Part, Part XXVI is announced to be "in a state of forwardness, and is fully expected to be ready for publication on the first of March;"—yet we have no news of its appearance.

The contributors to this work are some of the most distinguished anatomists and physiologists of Great Britain; and in addition to these we notice the names of Audouin, Breschet, Deshayes, Dutrochet, J. G. St. Hilaire, of Paris, Vrolik, of Amsterdam, and Wagner, of Erlangen.

2. The "*Cyclopædia of Practical Surgery*" is the only one of the works at the head of this article not now before us. Part XI, we believe, is the last. It contains Fistula, General Doctrine of Fracture, and an appendix on Reunion of Bone;—the first by the Editor, Dr. Costello, the second by Dr. J. Macdonnell, and the third by T. Wilkinson King, Esq., of Guy's Hospital. Amongst the collaborators are enrolled many of the most eminent surgeons of Great Britain and France; and although from the mode in which "it drags its slow length along," it is difficult to say when this Cyclopædia will be completed, it must be admitted, that many of the articles are of sterling value: one of them on Cancer, by Dr. Walshe, has been republished in this country by Dr. John Mason Warren, of Boston.

Both these Cyclopædias are illustrated by well conceived and well executed wood cuts, which add greatly to their value.

3. The "*Dictionary of Practical Medicine,*" by Dr.

Copland is in every respect an extraordinary work; wonderful to be executed wholly by one man. No single article can be referred to without the reader being astonished at the extent of information, and the classifying and condensing powers of the author. Dr. Copland is probably entitled to the credit of having first proposed such an encyclopædiac work on practical medicine in Great Britain. Cooper's Surgical Dictionary existed; and it was deemed important to have a similar work on medicine. In Pettigrew's "*Medical Portrait Gallery*," in a biographical notice of Dr. Copland, it is stated: "In 1825, Dr. Copland projected an 'Encyclopædiac Dictionary of the Medical Sciences,' and drew up a prospectus of the undertaking. In this he was to have been assisted by his friend Dr. Dunglison, now of the United States, and by the late Dr. Gordon Smith; and the work was actually agreed upon by Messrs. Underwood, medical publishers, when the panic of the period caused them to relinquish it."

This statement is correct in many particulars, but not in all. It contains a slight anachronism. Dr. Dunglison, we believe, left England in 1824; and in that year the project was conceived, and advertised in the "*London Medical Repository*," of which Drs. Copland and Dunglison were at the time editors. Mr. Henry Earle, of Bartholomew's Hospital, was to have prepared the surgical portion. This was probably the germ so ably developed by Dr. Copland, singly and alone, in his "*Dictionary of Practical Medicine*," the first part of which appeared in 1832. Part IX, the last published, concludes the second volume. It comprises the articles from Lungs, œdema of, to Ozœna inclusive. Two-thirds of the work are considered to be completed; but unless there be greater expedition in issuing the remaining parts than their predecessors, it will be yet six years before the learned author will see the term of his unrivalled efforts.

Of the "*Cyclopædia of Practical Medicine*" edited by Drs. Forbes, Tweedie and Conolly, and on this side of the Atlantic by Dr. Dunglison, we have spoken more than once. The English edition is completed; and the parts of the American edition are advertised to appear every fortnight. Nine parts have appeared already, and, we believe, punctually; so that the whole work will doubtless be in the hands of the subscribers in a year from the publication of the first part. Part IX contains:—Fungus Hæmatodes, Dr. Kerr: Galvanism, Dr. Apjohn and Dr. Dunglison: Gastritis, Dr. Stokes: Gastrodynia, Dr. Barlow: Gastro-enteritis, Dr. Stokes: Glanders, Dr. Dunglison: Glossitis, Dr. Kerr: Glottis, Spasm of the, Dr. Joy: Gout, Dr. Barlow: Hæmatemesis, Dr. Goldie: Hæmoptysis, Dr. Law: Headache, Dr. Burden: Heart, Diseases of, Dr. Hope: Heart, Dilatation of, Dr. Hope: Heart, Displacement of, Dr. Townsend: Heart, Fatty and Greasy degenerations of, Dr. Hope: Heart, Hypertrophy of, Dr. Hope.

So much for the English Encyclopædias.

Of the published French Medical Dictionaries we have no hesitation in placing the *Dictionnaire de Médecine* at the head. It is in its second edition: the first we have likewise before us. Volume first of the new edition was published in 1832: vol. 28th, this year. It contains the articles from *Sabine* to *Submersion*, inclusive; and when we name as their authors, Adelon, A. Bérard, Blache, Cazenave, Desormeaux, Guérard, Guersant, Lagneau, Murat, Ollivier, Orfila, Raige-Delorme, Richard, Rochoux, Soubeiran, and Velpeau, it is needless to say that they are ably executed. To those who desire an admirable encyclopædiac work in the French language, we most unfeignedly recommend this.

Of the *Dictionnaire des Etudes Médicales* we cannot help speaking with mortification. Four volumes only, from A to Del, published in 1838 and 1839, have been received by us; and to all inquiries of the Parisian booksellers, the reply is "the fifth volume has not yet appeared." It has unquestionably stopped altogether; for the article on Diabetes, by M. H. Bell, intended, if we mistake not, for the fourth volume, has been published in a separate form. We are mortified; partly because we have been deceived, and an incomplete work left on our hands; but mainly because several of the articles are excellent monographs of reference. It was conducted chiefly by the younger members of the profession, but did not on that account exhibit less talent or less zeal and industry. The articles in the fourth volume from *Cœur, Maladies du*, to *Délivrance* inclusive, are written by Letalenét, F. F. Francois, Caffe, Fée, H. Sestier, Sanson-Alphonse, P. Gentil, Videcoq, F. Sestier, A. Tavernier, A. Andral, Dupley, Bell, P. Guillemot, A. Férey-Demay, A. Lenoir, C. Denonvilliers, A. Juames, L. M. Michon, Sédillot, Ph. Rigaud, Martins, Rayor and Falret;—some of them not unknown to fame; but the greater part but little or not at all heard of among us.

It remains for us to allude briefly to the great German Encyclopædia; the first volume of which was published at Berlin in the year 1828, and the last, volume xxxi., in 1843. It was originally edited by C. F. von Gräfe, C. W. Hufeland, H. F. Link, K. A. Rodolphi, E. von Siebold; of whom one only, Dr. Link, survives. The present editors are D. W. H. Busch, J. F. Dieffenbach, J. F. C. Hecker, E. Horn, J. C. Jüngken, H. F. Link, J. Müller, all men of distinction, and some of them of world-renown. The last volume contains the articles *Schwangerschaft aussenhalb der Gibârmutter* ("Extra-uterine Pregnancy") to *Spätgeburt* ("Protracted Gestation") inclusive. The *Verzeichniss der Mitarbeiter* or list of Collaborators contains most of the distinguished names of Germany; and the authors of the articles in the 31st volume are Dommes, Ebert, Frank, Gedike, Hecker, Hertwig, W. Horn, Hüter, Ideler, Kersten, Langheinrich, Magnus, Von Schlechtendal, Schlemm, Schotte, G. Simon, Steinheim, Troschel, and Zabel. The individual contributions, as might be expected in so large a work, whether written by one person or by many, are unequal; and some of them very defective, yet it is a valuable addition to the library of the physician who is acquainted with one of the most important of the modern languages—the German.

These brief notices will be sufficient to show how actively the members of the profession are every where engaged in recording the results of ancient and modern observation,—and at the same time, how popular encyclopediac works on medicine have become as they have in other departments of science. It is convenient to have the whole body of a science condensed into a compass of even thirty volumes; and to one whose duty as a teacher, it is, to render himself acquainted with whatever has been done in his special department, they are all but indispensable.

*A Manual of Examinations upon Anatomy and Physiology, Surgery, Practice of Medicine, Chemistry, Materia Medica, Obstretrics, etc. Designed for the use of Students of Medicine throughout the United States.* By J. L. Ludlow, M. D. Philadelphia, Edward Barrington and George D. Haswell, 1844, pp. 615.

The title of this work explains its character and objects. The subjects which it embraces are discussed in the form of questions and answers, designed to recall to the mind of the student the important facts and principles which he may have read or heard in lectures. If used for that purpose only, such performances would scarcely be condemned by any one; but, when employed as substitutes for elaborate treatises on the various subjects of medical science, and as a sort of labor-saving machinery, by the aid of which every thing is to be memorized and repeated, parrot-like, instead of being investigated and understood, they are in the highest degree pernicious. In science, as in every thing else, nothing truly valuable is to be acquired without labor, and those who believe otherwise, find out, sometimes when it is too late, that they have been grievously mistaken. What the learner should desire to know, is, not how to avoid the labor of study, but how to employ the energies of his mind most advantageously.

Frequent examinations by a competent teacher, all must admit, are of the greatest importance to the student. By this means, not merely is his memory refreshed as to what he has learned, but his errors and misconceptions are corrected by the opportune remarks and explanations of the examiner.

We have glanced through Dr. Ludlow's work, and as far as we can judge by a hasty survey of its contents, it appears to be carefully written, both as it regards language and material facts. In this respect, we can cheerfully commend it to the notice of students of medicine, more especially those following the courses of instruction given in the University of Pennsylvania, for which, in fact, it appears to be specially adapted.

